# ProCOC: The prostate cancer outcomes cohort study

**DOI:** 10.1186/1471-2490-8-9

**Published:** 2008-06-17

**Authors:** Martin Umbehr, Thomas M Kessler, Tullio Sulser, Glen Kristiansen, Nicole Probst, Johann Steurer, Lucas M Bachmann

**Affiliations:** 1 Horten Centre for patient oriented research and knowledge transfer, University of Zurich, University Hospital of Zurich, Postfach Nord, 8091 Zurich, Switzerland; 2 Urological Clinic of the University Hospital of Zurich, Frauenklinikstrasse 10, 8091 Zurich, Switzerland; 3 Department of Pathology of the University Hospital of Zurich, Rämistrasse 100, 8091 Zurich, Switzerland; 4 The Cancer Registry of the Canton Zurich, Vogelsangstrasse 10, 8091 Zurich, Switzerland

## Abstract

**Background:**

Despite intensive research over the last several decades on prostate cancer, many questions particularly those concerning early diagnosis and the choice of optimal treatment for each individual patient, still remain unanswered. The goal of treating patients with localized prostate cancer is a curative one and includes minimizing adverse effects to preserve an adequate quality of life. Better understanding on how the quality of life is affected depending on the treatment modality would assist patients in deciding which treatment to choose; furthermore, the development of prognostic biomarkers that indicate the future course of the illness is a promising approach with potential and the focus of much attention. These questions can be addressed in the context of a cohort study.

**Methods/Design:**

This is a prospective, multi-center cohort study within the canton of Zurich, Switzerland. We will include patients with newly diagnosed localized prostate cancer independently of treatment finally chosen. We will acquire clinical data including quality of life and lifestyle, prostate tissue specimen as well as further biological samples (blood and urine) before, during and after treatment for setup of a bio-bank. Assessment of these data and samples in the follow up will be done during routine controls. Study duration will be at least ten years. Influence of treatment on morbidity and mortality, including changes in quality of life, will be identified and an evaluation of biomarkers will be performed. Further we intend to set up a bio-bank containing blood and urine samples providing research of various natures around prostate cancer in the future.

**Discussion:**

We presume that this study will provide answers to pertinent questions concerning prognosis and outcomes of men with localised prostate cancer.

## Background

Prostate cancer is the most frequent newly diagnosed malignancy in men in the Western world, and the second leading cancer-related cause of death. In the USA, 219,000 newly diagnosed cases and 27,000 related deaths have been predicted for 2007 [1]. This data is more clearly described by Walsh [2], who noted that approximately 1 of 6 men in the USA were diagnosed with prostate cancer, while 1 of 34 died from the cancer. Prostate cancer incidence rates continue to increase, although at a slower rate than reported in the early 1990s and prior. Nevertheless, given that the population in the developed world is rapidly aging, the percentage of cases of the disease is expected to rise dramatically. Taking this data into account, the medical and socio-economic consequences of prostate cancer are difficult to estimate.

Despite intensive research over the last several decades, many questions, particularly those concerning early diagnosis and the choice of optimal treatment for each individual patient, still remain unanswered. There are two main problems today.

PSA (prostate specific antigen) screening increases the number of patients suspected of cancer due to the high number of false positive results. Subsequent needle biopsies lead to over-diagnosis and lead to the detection of indolent cancers which would not have affected men in their lifetime [3-6]. Two trails are ongoing investigating the potential benefit due to PSA screening, completion is expected within the next five years [7,8]. Already now some authors questioned the clinical value of these studies. They stated that "it is uncertain whether these trials will have sufficient power to address the primary end point of reductions in deaths from cancer, because PSA screening of the control groups may occur outside these studies and definitive treatment with surgery or radiation therapy is not mandated [2]."

The second problem concerns the selection of appropriate treatment. Adequate choice is difficult because the natural course of disease is often unknown and both patient and clinician face the challenging decision whether active treatment is justified. Indeed, some studies showed a reduction in mortality [9-11]. However, identification which of the available treatments is best for an individual patient remains controversial, particularly because quality of life data are only limited available. Trials in this field are currently in progress. The Veterans Affairs Prostate Cancer Intervention vs. Observation (PIVOT) trial compares radical prostatectomy versus observation in 731 men and will complete the follow-up in 2009. The ongoing UK Prostate Testing for Cancer and Treatment Study (ProtecT) will randomize men with prostate cancer to receive radiation, undergo radical prostatectomy, or remain in observation; this trial is nested within a superior PSA screening trial, and study accrual is estimated to be completed in 2008.

The goal in treating patients with localized prostate cancer is a curative one and includes minimizing adverse effects to preserve an adequate quality of life. Better understanding on how the quality of life is affected depending on the treatment modality – surgery, radiation, active surveillance or even a palliative transurethral resection of the prostate (TUR-P) – would be a first, important step by assisting patients in deciding which treatment to choose. An improvement in the knowledge concerning prognosis would be a further, mostly important step. Here, the development of prognostic biomarkers that indicate the future course of the illness is a promising approach with potential and the focus of much attention. A further topic of interest concerning the prognosis of prostate cancer and prostate cancer survivors is the influence of lifestyle factors, such as nutrition and physical activity. Patients cured from cancer die of non-cancer causes at a higher rate than the general population and co-morbid conditions are probably the result of cancer treatment, genetic predisposition and lifestyle factors [12]. Increasing the knowledge concerning these topics could be of precious help in optimizing treatment outcome.

The purpose of this paper is to describe the rationale, the methodology and the design of a new cohort study. With this study we intend to increase the knowledge on optimal and individualized treatments of men with localized prostate cancer. We believe that its findings will contribute to a better quality of recommendations of physicians at the doctor patient encounter. Moreover, our genetics research branch will provide new insights in pathophysiology of prostate cancer and will have the potential to guide further clinical research particularly in prognostic testing.

## Methods/Design

### Study design

This is a prospective, multi-center cohort study with observational character concerning outcome. We will acquire clinical data including quality of life and lifestyle, prostate tissue specimen as well as further biological samples (blood and urine) before, during and after treatment for setup of a bio-bank.

### Study location

We set up this study within the Canton of Zurich, Switzerland. An expansion to a national or even international study is possible and intended.

### Partners

• Urological, Pathological and Radio-Oncological Units and Departments of the University Hospital of Zurich, the Cantonal Hospital of Winterthur and the City Hospital of Zurich.

• Cancer Registry of the Canton of Zurich.

• Department of Clinical Chemistry of the University Hospital of Zurich.

• Horten Center for patient orientated research and knowledge transfer of the University of Zurich.

### Study population

All men with newly diagnosed localized prostate cancer within the participating urological clinics are principally candidates for study participation. Treatment finally chosen is independent of study participation and *vice versa*. Exclusion criteria are unwillingness to sign the informed consent, an expected survival less than 6 month and referral to the participating urological clinics only for treatment due to intricate data and sample collection.

### Study sample size and duration

We performed a sample size calculation for survival endpoints based on the recommendation of Dupont and Plummer [13]. We have set the alpha error at 0.05 and the beta error at 0.2 and assumed a median survival time in the control group of 15 years. We specified a hazard ratio of the control group relative to the experimental group of 1.2, an accrual time during which patients are recruited of ten years and no additional follow-up time after end of recruitment. Since we are interested in various endpoints we chose an extreme ratio of control to experimental patients of 5 to be on the safe side. Based on these assumptions the required sample size is 1545. Given the size of the catchment's area we anticipate to include about two hundred new patients annually which lead to a sufficient number of patients to perform analyses after the end of the recruitment period.

### Procedure

**Figure 1:**
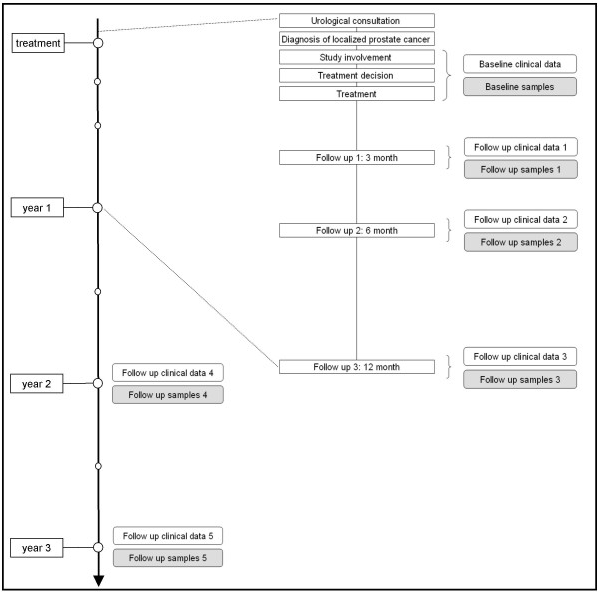
Study flow (the first three years).

We will recruit patients during urological consultations and after diagnosis of localized prostate cancer. Men who meet the inclusion criteria will receive detailed information about the study. In particular, the aims, methods and intension of setup a bio-bank will be fully explained and written informed consent will be obtained. Data and samples (prostate tissue, blood and urine) will be acquired during the routine consultation before, during and after the treatment. The follow up point of times are usually three, six and twelve month after treatment, then annually. Assistance by study nurses will ensure a mostly complete data acquisition. An overview of the study flow during the first three years is illustrated in figure 1. Unexpected events between the routine controls will be recorded and followed. Within the quality control all acquired data will become controlled before input into the central data base will take place. A reversible anonymization for all samples belonging to the bio-bank will be performed. In a preliminary phase of the study we will translate and validate the EPIC (Expanded Prostate Cancer Index Composite) questionnaire into German language to have a mostly precise instrument for assessing disease specific quality of life and especially changes in quality of life.

### Kind of collected data and samples

• Clinical data including quality of life and lifestyle factors. For general quality of life assessment we use SF-36v2 questionnaire and a "Feeling Thermometer", a valid and reliable global estimate of health-related quality of life. For prostate cancer specific quality of life we will use the EPIC questionnaire (Expanded Prostate cancer Index Composite) and the IPSS (International Prostate Symptoms Score). In a preliminary phase of the study we will perform a translation and validation of the EPIC questionnaire into German language.

• Prostate needle biopsy cores are already available due to preceded diagnosis and need only a special labeling. In case of prostatectomy as final treatment the whole organ will be stored, partly as formalin fixed and paraffin embedded specimen in the corresponding pathology unit of the hospital, where treatment is performed (multi-center storage), partly a shock frozen specimen in the central bio-bank of the Department of Pathology of the University Hospital of Zurich. The same procedure will be performed in case of repeated prostate needle biopsy.

• For the bio-bank we will acquire further one tube of whole blood, one tube of heparin full blood and two tubes of EDTA full blood, all of them as venous blood; further about 40 ml of native midstream urine. After anonymization these samples will be immediately send to the Clinical Chemistry of the University Hospital of Zurich, where aliquotation, DNA extraction, freezing and storage will take place.

### Outcome measures (baseline and follow up)

Data and samples acquired at diagnosis and before treatment belong to the baseline data. Data and samples acquired during the follow up and after the treatment belong to the follow up data. With exception of active surveillance as treatment or in case of suspicion of cancer recurrence, during the follow up no prostate tissue will be acquired routinely, whereas the clinical data and blood and urine samples for the bio-bank will be acquired at all the visits.

### Objectives

• To describe epidemiologic aspects of patients with newly diagnosed localized prostate cancer; in particular, to evaluate survival, functional outcomes, quality of life and life style in the context of treatment modality, prognosis and other clinical information.

• To assess the clinical relevance of recently proposed biomarkers.

• To develop individualized, science-based treatment recommendations for patients with localized prostate cancer.

• To link results from basic science to applied clinical research. For example, the study setup allows the investigation of promising prognostic genetic and post-genetic markers in a realistic clinical context and facilitates the translation of basic scientific discoveries into clinical practice and patient care.

• To provide a research platform for future diagnostic and prognostic markers using the systematically collected set of tissues and body fluids.

• To generate a German version of the EPIC questionnaire to get a validated instrument for assessment of quality of life in prostate cancer patients available for German speaking countries.

### Safety monitoring and adverse events

Any unexpected and/or adverse events will be recorded and followed until they have abated or until a stable situation has been reached. Treatments or interactions may necessary due to such events will take place within the urological or general physician's consultation.

### Ethics

This cohort study will be performed in accordance to the World Medical Association Declaration of Helsinki [14] and the guidelines of the Swiss Academy of Medical Sciences concerning scientific research involving human beings [15] and bio-banks [16] and will seek approval by the Ethical Committee of the Canton of Zurich. Further, all handling of personal data will strictly comply with the federal law of data protection of Switzerland (Bundesgesetz über den Datenschutz (DSG) vom 19. Juni 1992).

### Statistical analysis

Epidemiologic data and patients' descriptives available on continuous scales will be presented with medians, interquartile ranges or means and standard deviations as appropriate. Categorical data will be presented as rates and percentages.

Association of individual (independent) variables on the outcome variables will be reported using correlation coefficients. Results from univariate analysis will inform multivariate modeling.

Assessment of causal associations will be performed using multivariate models including potential confounders along with the independent variables of interest. Prognostic scores will be built using either multivariate logistic regression analysis or Cox proportional hazard models. Models will be validated in cross samples. Calibration and discrimination of the cross-validated prognostic instruments will be assessed using the Brier Score.

## Discussion

This paper describes the rational, methodology and design of a prospective, multi-center cohort study within the Canton of Zurich including patients with localized prostate cancer independent of treatment finally chosen. We intend to set up a prospectively acquired clinical database including quality of life data and lifestyle factors with corresponding prostate tissue specimens (biopsy cores and/or whole organ, dependent of treatment finally chosen) and further bio-samples (blood and urine).

Based on the results we will identify treatment specific influences on quality of life and formulate appropriate treatment recommendations. Examination of biomarkers will contribute to a better understanding of the underlying biological mechanisms and will guide the development of new diagnostic and prognostic algorithms. Therefore this study will help to improve decision-making. It will help patients weighing the pros and cons of various treatment modalities and it will help physicians to inform their patients adequately.

## Competing interests

The authors declare that they have no competing interests.

## Authors' contributions

All authors participated in study design; MU, LMB, TMK, GK, NP and JS drafted the protocol. TS critically reviewed the protocol; GK designed a common histo-pathology interpretation and storage guideline for prostate tissue samples; NP had consulting function in the setup and designing of the bio-bank; MU, LMB, TMK and JS obtained funding. All the authors read and approved the final manuscript.

## Pre-publication history

The pre-publication history for this paper can be accessed here:


